# Icon Familiarity Affects the Performance of Complex Cognitive Tasks

**DOI:** 10.1177/2041669520910167

**Published:** 2020-03-04

**Authors:** Zhangfan Shen, Linghao Zhang, Xing Xiao, Rui Li, Ruoyu Liang

**Affiliations:** School of Design, Jiangnan University, Wuxi, China

**Keywords:** familiarity, icon, complex cognitive task, working memory

## Abstract

The purpose of this study was to investigate whether and how users’ familiarity with symbols affects the performance of complex cognitive tasks which place considerable demands on working memory resources. We combined a modified math task paradigm with our previous icon familiarity training paradigm. Participants were required to complete a mathematical task involving icons to test their ability to perform complex cognitive tasks. The complexity of the task was manipulated using three independent variables: icon familiarity (high-frequency vs. low-frequency), whether or not the equation requires substitution (substitution vs. no-substitution), and the number of steps required for solution (one step vs. two steps). The results showed that participants performed better on the equation-solving task when it used icons they were more extensively trained on. Importantly, icon familiarity interacted with the complexity of the task and the familiarity effect on performance (accuracy and response time) became greater when the complexity increased. These findings provide evidence that familiarity affects not only the ease of information retrieval but also the ease of subsequent processing activities associated with these information, which extends our understanding of how familiarity affects working memory. Moreover, our findings have practical implications for improving interaction efficiency. Before the operators formally use a digital system, they need to learn the precise meaning of those complex or unfamiliar symbols in a certain context as much as possible.

As an important carrier of information, icons are widely used in digital interfaces, such as those of desktop computers, smartphones, mobile tablets, environmental monitoring systems, and multimedia systems in automobiles, serving as interpretive channels between users and information systems (Chi & Dewi, 2014; Li et al., 2017; X. Ma et al., 2015; Silvennoinen et al., 2017; SriLakshmi et al., 2017). Compared with text, graphic symbols have many advantages in conveying information. First of all, icons are able to transcend language barriers and convey information to people from different countries on many occasions, such as railway stations, airport, and other public places (Gittins, 1986; Horton, 1994). Besides, icons present semantic information in a condensed form (Blankenberger & Hahn, 1991; Rogers, 1989; Salman et al., 2012; Ziefle & Schröder, 2006). In other words, using graphical symbols to convey information is much more space-efficient than using text, which is especially crucial for digital interfaces with limited display space. Importantly, some researchers also reported that graphical symbols can often be recognized more accurately and quickly than relevant word equivalents (Arend et al., 1987; Muter & Mayson, 1986). For example, in Arend et al.’s study, a search-and-select paradigm was adopted to examine the effects of visual characteristics of icons for menu selection. They revealed that users in general identified icons faster than word commands. Similarly, in Muter and Mayson’s study, they compared menu selection for text-only menus to menu selection where the text was supplemented by graphics. The results showed that the addition of graphics reduced about 50% error rate with respect to text-only condition. However, to data, despite well-designed icons help users improve work efficiency and reliability (Horton, 1994), there is still a lack of guidance on icon design in the field of digital interface. The cognitive obstacles caused by poorly designed icons will hurt user experience and even result in major accidents in certain situations, such as interacting with on-board information system while driving. Therefore, it is of great significance to explore the design methods of icons used in digital interfaces.

Visual search is a common human activity which is acknowledged to be a perception task involving visual attention, attention shift, and scan of particular environment for a target (Xu & Yue, 2014). Treisman’s Feature Integration Theory (FIT) is one of the most influential and important theories of visual information processing in the last decades (Quinlan, 2003). FIT proposed two functionally independent and sequential processing stages: a parallel, preattentive first stage and a serial, second stage controlled by visual selective attention, suggesting that a limited set of attributes such as color, size, motion, and orientation could be processed in parallel, and serial deployments of visual attention were required to identify the target defined by two or more attributes (Treisman, 2006; Treisman & Gelade, 1980; Wolfe, 2007). Based on the FIT, Wolfe et al. (1989) proposed the Guided Search Theory and indicated that previous parallel stage was followed by an attentional bottleneck with a serial selection rule that then fed into parallel target recognition processes. The essential difference from the FIT is that the information from the first stage could be used to guide the deployments of selective attention in the second stage (Wolfe, 2007; Wolfe & Horowitz, 2004; Wolfe et al., 2011). Moreover, Duncan and Humphreys (1989) claimed that there were two essential factors determining visual search performance: the similarity of the target to the distractors and the similarity of the distractors to one another. According to their Attentional Engagement Theory, visual search difficulty is directly related to the former and inversely related to the latter.

Since targets are rare in real dynamic scenes (e.g., medical or airport screening), researchers usually simplify the environment and design-specific task to ask participants to locate a certain target in a field of similar distractors to test a person’s visual sharpness and mental response time (RT; Epelboim et al., 1995; Võ & Wolfe, 2013). In graphical symbol design and research areas, a series of visual search studies were carried out to explore the effects of design properties of icons on users’ cognitive performance. McDougall et al. (2000) conducted experiments to investigate the factors considered central to icon usability, revealing that users performed better when using simple visual icons than complex visual icons. Stammers and Hoffman (1991) found that users identified concrete icons more efficiently than abstract icons. Goonetilleke et al. (2001) and McDougall et al. (2001) showed that icons with closer semantic distances were easier to understand. According to previous studies, three characteristics need to be considered when designers create an icon: visual complexity, which refers to the amount of detail in an icon; concreteness, which refers to the degree to which an icon depicts object in the real world; and semantic distance, which indicates the closeness of the relationship between an icon’s visual representation and its intended meaning (S.-C. Huang et al., 2015; McDougall et al., 1999; Silvennoinen et al., 2017; Stevens et al., 2009). However, some researchers also noticed that the differences in performance among different kinds of icons diminished if users became familiar with those icons (Green & Barnard, 1990; Stotts, 1998). Thus, Isherwood et al. (2007) defined icon familiarity as the frequency of use, which relates directly to experience with an icon, and they believed that this form of familiarity could be trained by presenting participants with icons over a number of blocks of trials. Subsequent experimental findings of icon familiarity showed that the importance of icon characteristics changed with user experience (McDougall et al., 2016).

Recently, to further explore this issue, we experimented with an icon visual search task across several days to simulate the effects of gaining increasing experience (Shen et al., 2018). After manipulating the participants’ level of familiarity with different icons, their ability to recall corresponding semantic information when cued with different icons was tested. The results showed that participants performed significantly better on both the visual search task and the semantic recall task when the icons were more familiar, and the beneficial effects of familiarity were larger when the icons were complex. These observations indicated that familiarity should be another key factor that has lasting effects on icon cognition. However, in the field of human–computer interaction, most previous studies on icon familiarity have focused on visual search tasks and recall tasks (Gittins, 1986; K. C. Huang, 2008; McDougall et al., 2000, 2006). Since the interaction between users and the visual elements of the interface is a comprehensive and complex process that includes a series of perceptions, comprehension, decision making, and reactions, there is a great need to investigate whether and how icon familiarity affects the tasks requiring higher order cognition that people must continuously retain multiple kinds of relevant information and intermediate results in working memory while simultaneously processing this information using complex techniques. Therefore, this study aims to explore the influence of icon familiarity on complex cognitive tasks and provide more experimental evidence to help graphical user interface designers improve the icon design process.

Previous work has shown that working memory is the system that is responsible for holding mental representations available for processing and temporary storage, which plays a key role in human cognition, especially for higher order cognition such as problem-solving, reading comprehension, reasoning, mental calculation that goes beyond immediate sensations, and memories (A. Baddeley, 2012; A. D. Baddeley & Hitch, 1974; Cassimatis et al., 2010; Lépine et al., 2005). One of the most important features of working memory is its limited capacity (Halford et al., 2007; Oberauer, 2009). In recent decades, exploring the cognitive mechanism of the limited working memory capacity has been a hot topic in cognitive psychology research. Hypotheses about what limits working memory capacity can be summarized into three principle theories: decay theory, interference theory, and resource-based theory (A. Baddeley, 2012; Oberauer et al., 2016). The decay theory suggested that the currently active representations in working memory decay over time, and this information would be unavailable for future processing if it is not reactivated within a certain timeframe (A. D. Baddeley et al., 1975; Camos et al., 2009; Schweickert & Boruff, 1986). In contrast, the interference theory assumes that representations in working memory do not decay on their own but that attempting to hold a large amount of active information in working memory’s limited capacity results in interference (Oberauer & Kliegl, 2006; Oberauer et al., 2012). Moreover, the resource-based theory indicates that an individual’s limited working memory resources need to be shared by the representations that are simultaneously being held available and being processed can easily account for our results both in substitution and no-substitution conditions (W. J. Ma et al., 2014; Oberauer et al. 2012). Specifically, both information processing and information storage activities are competing for the same limited resources within working memory.

Based on these working memory theories, researchers have carried out numerous studies to investigate the connection between working memory and higher order cognition (Beilock et al., 2004; Bull et al., 2008; Fung & Swanson, 2017; Swanson et al., 2004). One suitable domain is mathematical problem-solving, for example, Anderson et al. (1996) adapted the Carlson et al.’s (1989) paradigm to an algebraic equation solving task to investigate the consumption of working memory resources during complex cognitive tasks. They controlled the complexity of the task by manipulating the number of transformations (one step or two steps) and whether participants were required to access the memory set. The results showed that performance was worse in the math task when the equations were more complicated. It is obvious that working memory plays a crucial role in this process. Similarly, according to resource-based theory, we can assume that when an icon is familiar, people need to use fewer working memory resources to retain and recall the icon and its associated semantic information in working memory. If familiarity with the stimuli determines the amount of working memory resources that is required for their manipulation, then familiarity should affect higher order cognition. Thus, in this case, we believe that icon familiarity should affect not only performance on visual search and recognition tasks but also performance on higher order cognitive tasks that consume more working memory resources.

Therefore, in this study, we tried to combine the math task paradigm (Anderson et al., 1996) with our icon familiarity training paradigm. To explore our prediction, we first experimentally controlled the familiarity of icons with a semantic recognition task. As in our previous study, we manipulated icon familiarity using high or low (10:1 ratio) exposure frequency during training sessions. Then, participants were required to complete a mathematical task that involved high- or low-familiarity icons to test their ability to perform complex cognitive tasks. We manipulated the complexity of the mathematical task by changing the substitution condition (substitution or no-substitution) and the number of steps (one step or two steps). The key problem was whether the difference in familiarity between icons would lead to differences in performance during the mathematical equation solving process. If our prediction is correct, then we should observe better performance on higher order cognitive tasks when the icons involved are more familiar. Moreover, we would also expect that as the complexity of the equations increases, the effect of icon familiarity on performance should also increase.

## Method

### Equipment and Participants

All the experiments were carried out in Ergonomics laboratory, Jiangnan University under normal office lighting (∼300 lux). Stimulus presentations and response collections were performed using a custom experimental program built in Unity 3D. Stimuli were displayed on a 17 in. (43 cm) LED monitor at a resolution of 1,280 × 1,024 and a refresh rate of 60 Hz. The viewing distance used was 50 cm.

Twenty college students (8 men and 12 women, ages ranging from 20 to 25 years, mean = 23.6) from Jiangnan University participated in this study. All participants completed and signed an informed consent form approved by the university institutional review board before participating in the experiments and they had never participated in similar experiments before. Participants were compensated a minimum of ¥50 and could receive a bonus payment based on performance ranging from ¥25 to ¥50.

### Materials

We selected graphical symbols from computer and smart phone systems for reference, and then revised and created more than 300 icons for the following experiments. The method and instructions we used were similar to those adopted in previous studies (McDougall et al., 1999, 2000; Shen et al., 2018). In this study, each icon was assigned a single Chinese word that five experts (experienced icon designers) agreed to be the best word label to represent the meaning of the icon. To ensure that the icon–word pairs for the following experiments were at the same level, we recruited 20 volunteers to rate the familiarity and the semantic distance of each icon–word pair in a 5-point scale. As described in the Introduction section, familiarity reflects the frequency with which items are encountered, and icons are to be regarded as familiar if they often appear in daily life (1 = *very unfamiliar*, 5 = *very familiar*). Semantic distance is a measure of the closeness of the relationship between icon and the meaning it represents (1 = *very not closely related*, 5 = *closely related*). If the semantic distance is not closely related, people can hardly identify the meaning of the icon when they first see it (Silvennoinen et al., 2017). Icon–word pairs were presented to them by Microsoft PowerPoint. Each page contained 10 icon–word pairs, and alongside each pair was a 5-point rating scale. For each volunteer, the order of icons and pages was random. According to the rating results, we eliminated 112 icon–word pairs of which the semantic distance were extremely close or far way and got 268 pairs with balanced ratings (*M* = 3.25, *SD* = 0.47). Subsequently, volunteers were required to rate the familiarity of those remaining pairs (1 = *very unfamiliar*, 5 = *very familiar*). Similarly, icon–word pairs which were too familiar or unfamiliar were excluded, and 80 icon–word pairs were finally selected (*M* = 2.87, *SD* = 0.31). Finally, one-way analyses of variance (ANOVAs) were conducted to ensure that all the 80 icon–word pairs selected were at the same level of semantic distance and familiarity. The results indicated that there was no significant difference among the semantic distance ratings, *F*(79, 1520) = 1.68, *p* = .25, or the familiarity ratings of these icons, *F*(79, 1520) = 1.28, *p* = .22. Thus, we successfully generated the materials needed for the following experiments.

### Semantic Recognition Task

**Figure 1.**
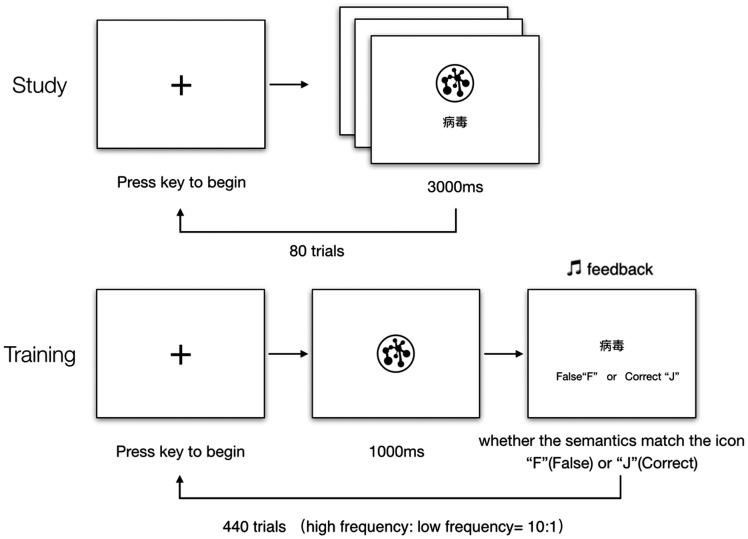
Trial Sequence for Semantic Recognition Task.

Firstly, participants learned the relationships between 80 icons and associated semantic information one-by-one in a random order. Subsequently, they performed a semantic recognition task across two training sessions, and each session consisted of 440 trials (see Figure 1). For each participant, there were 80 icons; half of the icons were randomly assigned to the high-frequency group, and the remaining half were assigned to the low-frequency group. As the ratio of high- versus low-frequency icons was controlled to be 10:1, we had 400 high-frequency trials and 40 low-frequency trials, with a total of 440 trials. Each trial began with the presentation of a fixation cross, and participants had to press any button to continue. A randomly selected icon was shown in the center of the screen for 2 seconds, which was followed by a screen showing semantic information. Participants had to respond based on whether the semantic information was associated with the icon that was shown on the previous screen. After participants pressed “F” (False) or “J” (Correct), they received auditory feedback that indicated whether their response was right or wrong. A 5-minute break was given during each experimental session. The trial order and whether the semantic information was associated with the icon in each trial were randomly determined for each participant and session. We considered accuracy and RT when reporting whether the semantic information was associated with the icon as dependent variables.

### Mathematical Problem-Solving Task

**Figure 2.**
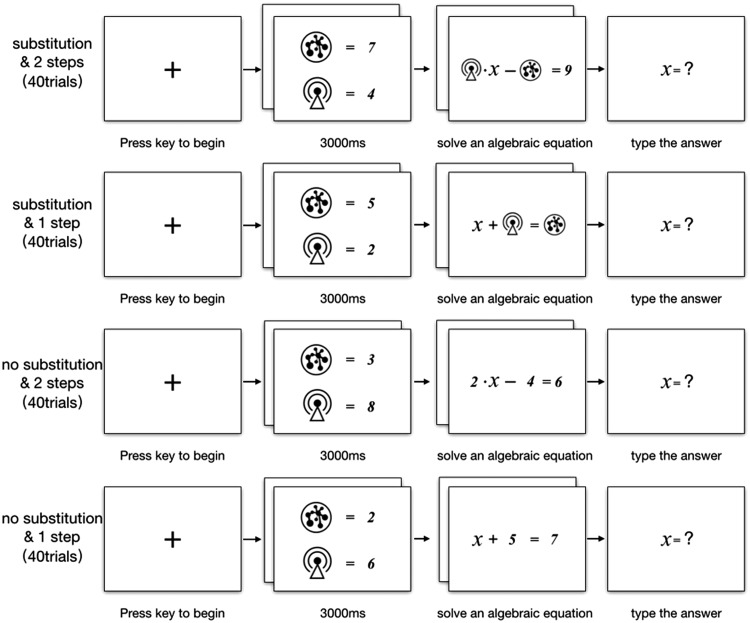
Trial Sequence for Mathematical Solving Task.

After the participants had become familiar with 80 icon–word pairs across two training sessions, with half of the icon–word pairs being presented more frequently, a mathematical problem-solving task was carried out to test participants’ ability to solve complex cognitive tasks. The task consisted of 160 different mathematical equations with one unknown value *x.* All equations were generated with the following constraints: (a) constants in equations were single digits and (b) the final value of *x*, which needed to be calculated, was an integer from −9 to 9. Participants were required to use addition, subtraction, multiplication, or division to calculate the unknown value *x.* Each experimental trial began with the presentation of a fixation cross, and participants had to press any button to continue (see Figure 2). Following the fixation cross, two icons from the same frequency group and two different digits were presented on the screen for 3 seconds. To solve the equation in the next step, participants had to encode and hold the icon–digit associations in working memory. After viewing the icons and digits, participants were shown an algebraic equation to solve in their heads and were given an unlimited amount of time. Pen, paper, or calculator was not provided during the test. Participants were required to press any button to type in their answer once they had calculated the value of *x*.

We used a 2 × 2 × 2 within-subjects design, with the independent variables of icon frequency (high vs. low), whether the equation required substitution with the corresponding digits (substitution vs. no-substitution), and whether the equation required one or two steps. Icons used in the mathematical problem-solving task were the same as those used in the semantic recognition task. For each participant, there were 40 substitution and two-step equations, 40 substitution and one-step equations, 40 no-substitution and two-step equations, and 40 no-substitution and one-step equations, with a total of 160 experimental trials. Regardless of whether there was substitution, participants first learned the icons and the associated digits. In all trials, half of the equations contained high-frequency icons, and the other half contained low-frequency icons. For each trial, both icons were from the same frequency condition. The trial order was randomly determined for each participant. We measured the participants’ accuracy and RT when solving the algebraic equations.

## Results

RT was analyzed by linear mixed-effects regression, and accuracy data were analyzed via logistic mixed-effects regression (Baayen et al., 2008; Jaeger, 2008). For the RT analyses, we considered only correct trials (11.2% error for the semantic recognition task; 7.6% error for the mathematical solving task). Afterwards, we exclude from the analyses cases with RTs greater than three median absolute deviations above or below the median RT, calculated separately for each participant and condition (10.1% for the semantic recognition task; 8.6% for the mathematical solving task).

### Semantic Recognition Task

**Figure 3.**
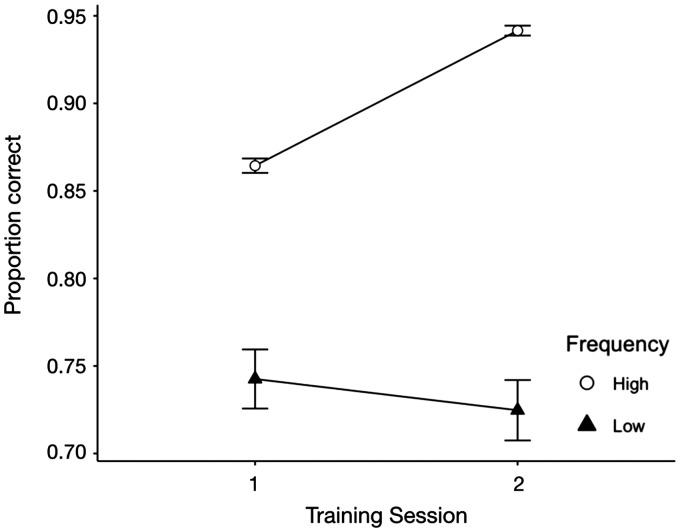
Mean Performance (Accuracy) on Semantic Recognition Task Trials for High-Frequency and Low-Frequency Icons Over Two Sessions of Training. *Note*. Error bars indicate ± 1 standard errors.

Figure 3 shows the accuracy data on semantic recognition task trails for high-frequency and low-frequency icons over two sessions of training. Participants recognized high-frequency icons more accurately than low-frequency icons, ΔAIC (akaike information criterion) = −291, LLR (log likelihood ratio) χ^2^(1) = 293.621, *p* < .001. For high-frequency icons, participants performed better in the second session than in the first session, ΔAIC = −246, LLR χ^2^(1) = 247.72, *p* < .001. However, there was no significant difference in the accuracy of recognizing low-frequency icons between the first and the second session, ΔAIC = 1.5, LLR χ^2^(1) = 0.497, *p* = .481. In addition, there was a significant interaction between icon familiarity and training session on accuracy, ΔAIC = −52.5, LLR χ^2^(1) = 54.572, *p* < .001.

**Figure 4.**
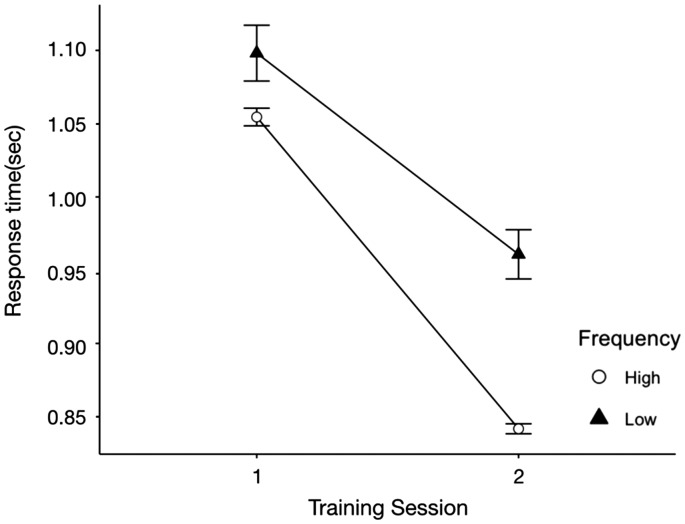
Mean Performance (RT) on Semantic Recognition Task Trials for High-Frequency and Low-Frequency Icons Over Two Sessions of Training. *Note*. Error bars indicate ± 1 standard errors.

Figure 4 shows the RT data on semantic recognition task trails for high-frequency and low-frequency icons over two sessions of training. Participants recognized high-frequency icons faster than low-frequency icons, ΔAIC = −67.1, LLR χ^2^(1) = 69.103, *p* < .001. For high-frequency icons, participants performed better in the second session than in the first session, ΔAIC = −164.5, LLR χ^2^(1) = 166.56, *p* < .001. However, there was no significant difference in the RT of recognizing low-frequency icons between the first and the second session, ΔAIC = −0.01, LLR χ^2^(1) = 2.015, *p* = .156. Besides, there was also a significant interaction between icon familiarity and training session on RT, ΔAIC = −8.3, LLR χ^2^(1) = 10.273, *p* < .001.

### Mathematical Solving Task

**Figure 5.**
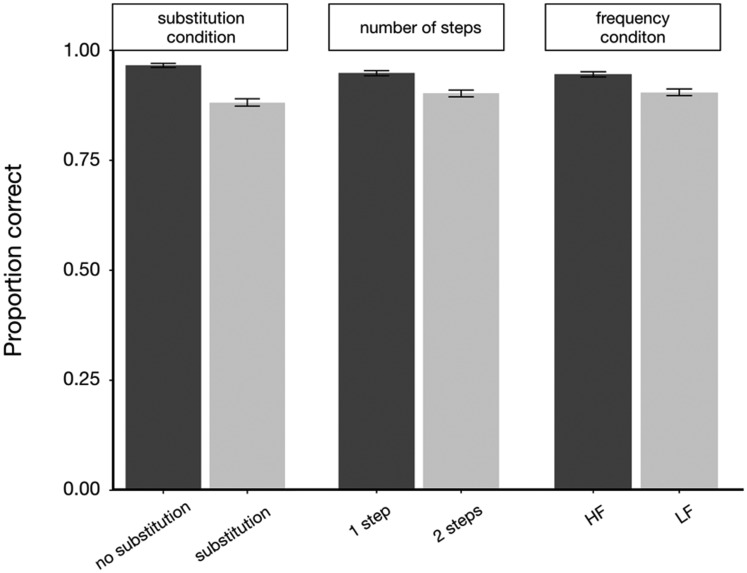
Mean Performance (Accuracy) on Mathematical Solving Task Trials in Different Conditions. *Note*. Error bars indicate ±1 standard errors. HF = high frequency; LF = low frequency.

Figure 5 shows the accuracy data on mathematical solving task trails in different conditions. Participants performed significantly better in no-substitution condition, ΔAIC = −80.7, LLR χ^2^(1) = 82.626, *p* < .001. In addition, performance declined significantly when the number of steps increased from one step to two steps, ΔAIC = −22.6, LLR χ^2^(1) = 24.603, *p* < .001. Importantly, participants solved the algebraic equations more accurately when the icons that they had to remember were more familiar, ΔAIC = −17.3, LLR χ^2^(1) = 19.480, *p* < .001.

**Figure 6.**
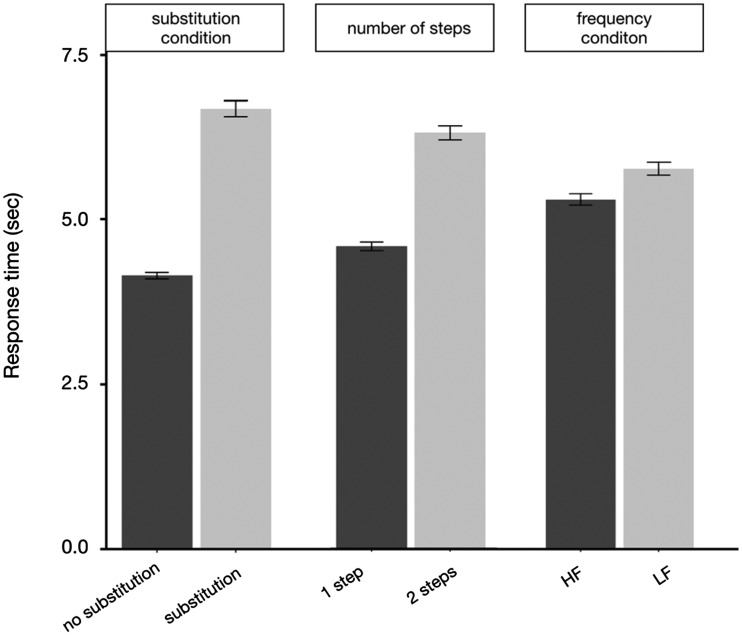
Mean Performance (RT) on Mathematical Solving Task Trials in Different Conditions. *Note*. Error bars indicate ±1 standard errors. HF = high frequency; LF = low frequency.

Figure 6 shows the RT data on mathematical solving task trails in different conditions. Participants also performed significantly better in no-substitution condition, ΔAIC = −304, LLR χ^2^(1) = 306.718, *p* < .001. Besides, participants needed more time to solve the equations when the number of steps increased, ΔAIC = −805.9, LLR χ^2^(1) = 807.984, *p* < .001, and they solved equations significantly faster when the icons were familiar, ΔAIC = −36, LLR χ^2^(1) = 38.011, *p* < .001.

**Figure 7.**
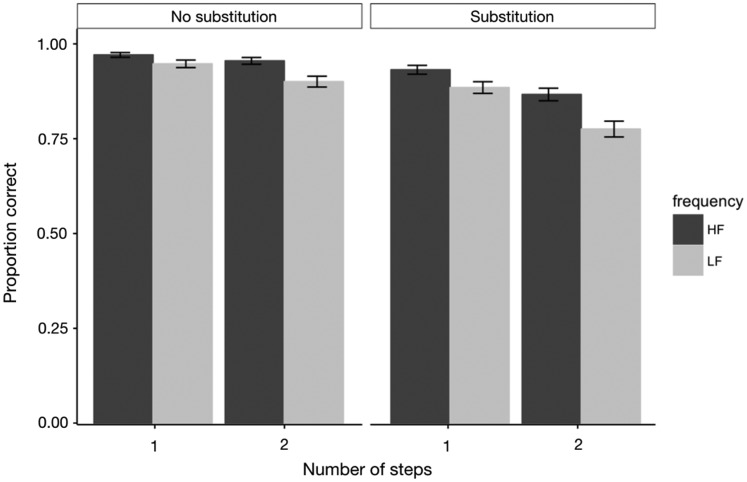
Mean Performance (Accuracy) on Mathematical Solving Task Trials for HF and LF Icons. *Note*. Error bars indicate ± 1 standard errors. HF = high frequency; LF = low frequency.

Figure 7 shows the accuracy data on mathematical solving task trials for high-frequency and low-frequency icons in different conditions. The difference in accuracy between high-frequency icons and low-frequency icons was significant in no-substitution and one-step condition, ΔAIC = −2.27, LLR χ^2^(1) = 4.273, *p* < .05; no-substitution and two-step condition, ΔAIC = −8.83, LLR χ^2^(1) = 10.838, *p* < .001; substitution and one-step condition, ΔAIC = −4.34, LLR χ^2^(1) = 6.336, *p* < .05; and substitution and two-step condition, ΔAIC = −10.06, LLR χ^2^(1) = 12.062, *p* < .001. Importantly, in addition to the main effects, there were two strong interactions. The effect of icon familiarity on accuracy was larger in the substitution condition than in the no-substitution condition, ΔAIC = −94, LLR χ^2^(1) = 99.957, *p* < .001, and it was also larger in the two-step condition than in the one-step condition, ΔAIC = −62.7, LLR χ^2^(1) = 68.666, *p* < .001. In other words, the effect of icon familiarity on accuracy significantly increased as the algebraic equations became more complex.

**Figure 8.**
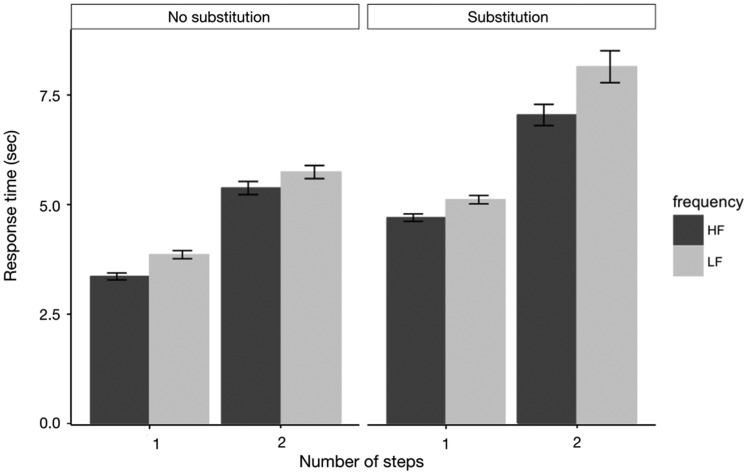
Mean Performance (RT) on Mathematical Solving Task Trials for HF and LF Icons. *Note*. Error bars indicate ± 1 standard errors. HF = high frequency; LF = low frequency.

Figure 8 shows the RT data on mathematical solving task trials for high-frequency and low-frequency icons. The effects of icon familiarity on equation solving speed were significant in no-substitution and one-step condition, ΔAIC = −22.21, LLR χ^2^(1) = 24.111, *p* < .001; no-substitution and two-step condition, ΔAIC = −3.55, LLR χ^2^(1) = 5.546, *p* < .05; substitution and one-step condition, ΔAIC = −14.01, LLR χ^2^(1) = −2.85, *p* < .05; and substitution and two-step condition, ΔAIC = −9.14, LLR χ^2^(1) = 11.131, *p* < .001. Besides, we also found significant interactions between icon familiarity and substitution condition, ΔAIC = −348, LLR χ^2^(1) = 353.96, *p* < .001, and between icon familiarity and number of steps, ΔAIC = −708.3, LLR χ^2^(1) = 714.31, *p* < .001. These strong interactions were consistent with what we observed in the analysis of accuracy data. In summary, as we predicted, icon familiarity interacted with the complexity of the task and the familiarity effect on performance (accuracy and RT) became greater when the complexity increased.

## Discussion

### Semantic Recognition Task

Most previous studies on the effect of familiarity have employed stimuli with preexisting differences in familiarity (Blalock, 2015; Cowan et al., 2015; McDougall et al., 2016; Siedenburg & McAdams, 2017). For example, McDougall et al. (2016) used preexisting icons as stimuli, dividing them into familiar and unfamiliar groups based on participants’ ratings and found that this varied considerably between individuals. In such cases, if variables are not controlled or are inadequately controlled, orthogonal factors may influence the result that is thought to be measured. However, in this study, we created and screened 80 icons at the same level, and these icons were also verified to have no significant differences in their level of familiarity or semantic distance using ANOVA. Then, we differentially trained participants on these icon–word pairs across two training sessions, exposing participants to half of the icons 10 times more often than the other half. The results of the mean performance on the semantic recognition task showed that participants performed significantly better when the icons were in the high-frequency group (see Figures 3 and 4), and there were significant interactions between icon familiarity and training session on both accuracy and RT. Besides, we can also notice that for both accuracy and RT data, the differences between high frequency (familiar) icons and low frequency (unfamiliar) icons became much greater as the training session increased. Thus, we successfully manipulated participants’ familiarity with icons and created familiarity differences by the end of the semantic recognition task.

In addition, we also found that the RTs for high-frequency and low-frequency icons significantly decreased throughout the training session, while the accuracy of low-frequency icons did not improve, remaining basically the same. The reason for this, we contend, is that with the increase in the number of trials, participants became more experienced with the task; thus, the RT required to complete each trial was significantly reduced. Meanwhile, since high-frequency icons appeared more frequently, participants had more opportunities to encode and rehearse icon–semantic associations through continuous testing, resulting in higher accuracy.

### Mathematical Problem-Solving Task

Numerous researchers have indicated that item familiarity (e.g., word frequency) affects the performance of working memory in visual search, recognition, and free and cued recall tasks (Clark, 1992; Cox et al., 2018; MacLeod & Kampe, 1996; Nelson & Shiffrin, 2013). In this study, we combined our familiarity paradigm with a modified algebraic paradigm in Anderson et al. (1996). Instead of requiring participants to hold a set of digits in working memory while trying to solve an mathematical equation, we presented them with two icons from same frequency group that were each associated with a single digit before the mathematical equation was presented. The results showed that both whether or not the equation requires substitution (substitution vs. no-substitution), and the number of steps required for solution (one step vs. two steps) had significant impacts on participants’ performance of solving equations. When icons appeared in the equations, participants had to first recall associated digits of different icons and then replace icons with those digits for calculation. We contend that these more involved tasks required more working memory resources and therefore imposed a greater degree of cognitive load on participants, which led to a low-level accuracy rate and long RT. Similarly, compared to the equations that only required one step to solve, we argue that the equations involving two steps might be much harder for the participants to calculate in their heads, which would consume more resources, resulting in worse performance. Importantly, the results presented here also revealed that participants’ familiarity with icons (icon–semantic associations) definitely influenced the performance on complex cognitive tasks, suggesting that familiarity affects not only the ease of information retrieval but also the ease of subsequent processing activities associated with these information, which extends our understanding of how familiarity affects working memory. In addition, the results shown in Figures 5 and 6 also indicated that, compared with the factors of transformation steps and icon familiarity, whether or not the equation required substitution of digits influenced participants’ performance most, implying that two different substitution conditions varied greatly in the demand for working memory resources. However, despite the impact of icon familiarity on participants’ performance was significant as well, the difference of working memory demanding was relatively small comparing with other two factors.

As described in the introduction, current working memory theories could be generally summarized into three cases: decay-based, interference-based, and resource-based theories (A. Baddeley, 2012; Oberauer et al., 2016). The decay-based theory suggests that working memory representations decay rapidly over time, and that decay can be counteracted by continuous rehearsal. From this point of view, we contend that unfamiliar icons and their associated digits might be relatively difficult to reactivate, as they would begin to decay faster than familiar icons after the coding phase, resulting in impaired mathematical performance in substitution conditions. This explanation seems reasonable; however, importantly, our results also showed that participants’ performance using familiar icons was still significantly better than that using unfamiliar icons in the no-substitution condition. Although participants encoded and retained the icons and corresponding digits, they did not need to use this information in the following equation solving phase. Obviously, the decay-based theory can only partially explain our results. On the contrary, the interference-based theory assumes that people’s ability to simultaneously hold several representations is limited by mutual interference between these representations (Oberauer & Kliegl, 2006; Oberauer et al., 2012). Specifically, interference was generated by the confusion among different item representations, and the interference by confusion arises from a retrieval mechanism called competitive queuing which describes retrieval from working memory as a competition among a number of retrieval candidates (Hurlstone et al., 2014; Lewandowsky & Farrell, 2008). The more a representation is activated, the more likely it is to be retrieved. Therefore, according to this theory, we contend that at the learning stage, the relationship between familiar icons and their associated digits was better encoded, and thus, the representations were stronger and less susceptible to interference. In contrast, the weak relationship between unfamiliar icons and associated digits was more likely to interfere with each other, which made it more difficult to recall correct digits and substitute corresponding icons in the equations. However, similar to the decay-based theory, it is still difficult to explain the familiarity effect that we observed in the no-substitution condition.

The resource-based concepts share a set of assumptions. (a) The pool of resources available for working memory tasks is limited and will be consumed eventually (Case et al., 1982). (b) Cognitive functions (e.g., holding representations available) and the processes of transforming or manipulating information share the same limited resources at the same time (Alloway et al., 2006; Logie, 2011). (c) The resources can be allocated flexibly to different tasks, and the performance of cognitive process increases monotonically with the resource amount allocated to it (Oberauer et al., 2016). Moreover, researchers also demonstrated that the more difficult the task is, the more resources are needed (Cowan et al., 2012; Luck & Vogel, 2013; Salthouse, 1992). For example, Salthouse (1992) carried out several experiments to test adults’ cognitive performance by manipulating task complexity. Results showed that complex cognitive tasks place much greater demands on a working memory resource. Therefore, we can assume that if the information is easy to encode and store, the resource demand for encoding and storage activities will be lower, and the information processing activities will have access to more available resources, leading to better information processing results. The data presented on the right side of Figures 7 and 8 demonstrate our hypothesis, showing that the performance in trials using the high-frequency icons was obviously better in the substitution condition. In line with the resource-based theory, we contend that since icon–digit associations with high familiarity consumed fewer working memory resources, there were more remaining working memory resources that could be allocated for solving equations, which resulted in better performance. Importantly, when the equations became more difficult (from one step to two steps), we observed a significant interaction between the familiarity effect and equation complexity. In other words, the familiarity effect was magnified when more working memory resources were required to process information.

In addition, we found that the familiarity effect remained even when participants did not need to replace the icons with digits in the mathematical problem-solving task. The reason for this, we believe, is that participants did not know whether the following equations would contain icons when they learned the icon–digit associations. Therefore, to correctly solve the equations, they did their best to encode and retain the icon–digit associations, which consumed certain working memory resources. The data presented on the left side of Figures 7 and 8 confirm this idea, illustrating that unfamiliar icons and their associated digits indeed consumed more working memory resources, which likely accounts for the participants’ impaired performance in the no-substitution condition.

## Conclusions

Previous studies have demonstrated that users’ icon familiarity plays an important role in cognitive performance on visual search and recognition task. However, does this kind of familiarity effect also affect higher order cognitive tasks? This study suggests that the answer is yes. In this article, we revealed that participants were significantly better at solving mathematical problems using familiar icons than unfamiliar icons and this beneficial effect of icon familiarity increased as the complexity of the cognitive task increased. In line with the resource-based theory, we conclude that (a) the encoding or binding of stimuli depends on a limited pool of working memory resources; (b) these operations consume more working memory resources when the stimuli are less familiar; and (c) maintaining or manipulating less familiar information results in less working memory resource available for performing more complex cognitive process. In addition, our findings have practical implications for improving interaction efficiency. Before the operators formally use a digital system, they need to learn the precise meaning of those complex or unfamiliar symbols in a certain context as much as possible. For example, in complex situational awareness systems such as radar interfaces or command platforms, the improvement of user’s familiarity with various graphic symbols will be greatly beneficial to the judgment, calculation and processing of comprehensive information.

## Limitations

Although this study provides some evidence for the familiarity effect on complex cognitive tasks and extends our understanding of how item familiarity influences working memory, it has several limitations. The target icons used in our study were selected and modified from current computer and smart phone systems. However, novel icons or graphical symbols that users have never seen before could remove the noises of the existing semantics and familiarity, and even simplify the experimental procedure. In addition, although the results of semantic recognition task showed that we had successfully manipulated the familiarity of icon–word pairs, we believe that an appropriate increase in the number of training session could lead to a more direct celling effect of high-frequency icons in the semantic recognition task and make the familiarity effect on the mathematical problem-solving task more obvious.

## References

[bibr1-2041669520910167] Alloway T. P. Gathercole S. E. Pickering S. J. (2006). Verbal and visuospatial short-term and working memory in children: Are they separable? Child Development, 77, 1698–1716. 17107455 10.1111/j.1467-8624.2006.00968.x

[bibr3-2041669520910167] Anderson J. R. Reder L. M. Lebiere C. (1996). Working memory: Activation limitations on retrieval. Cognitive Psychology, 30, 221–256. 8660785 10.1006/cogp.1996.0007

[bibr4-2041669520910167] Arend U. Muthig K. P. Wandmacher J. (1987). Evidence for global feature superiority in menu selection by icons. Behaviour and Information Technology, 6, 411–426.

[bibr5-2041669520910167] Baayen R. H. Davidson D. J. Bates D. M. (2008). Mixed-effects modeling with crossed random effects for subjects and items. Journal of Memory and Language, 59, 390–412.

[bibr6-2041669520910167] Baddeley A. (2012). Working memory: Theories, models, and controversies. Annual Review of Psychology, 63, 1–29. 10.1146/annurev-psych-120710-100422 21961947

[bibr7-2041669520910167] Baddeley A. D. Hitch G. J. (1974). Working memory In Bower G. H. (Ed.), Recent advances in learning and motivation (Vol. VIII, pp. 47–90). Academic Press.

[bibr8-2041669520910167] Baddeley A. D. Thomson N. Buchanan M. (1975). Word length and the structure of memory. Journal of Verbal Learning & Verbal Behavior, 14, 575–589.

[bibr9-2041669520910167] Beilock S. L. Kulp C. A. Holt L. E. Carr T. H. (2004). More on the fragility of performance: Choking under pressure in mathematical problem solving. Journal of Experimental Psychology: General, 133, 584–600. 15584808 10.1037/0096-3445.133.4.584

[bibr10-2041669520910167] Blalock L. D. (2015). Stimulus familiarity improves consolidation of visual working memory representations. Attention, Perception, & Psychophysics, 77, 1143–1158. 10.3758/s13414-014-0823-z 25720758

[bibr11-2041669520910167] Blankenberger S. Hahn K. (1991). Effects of icon design on human-computer interaction. International Journal of Man-Machine Studies, 35, 363–377.

[bibr12-2041669520910167] Bull R. Espy K. A. Wiebe S. A. (2008). Short-term memory, working memory, and executive functioning in preschoolers: Longitudinal predictors of mathematical achievement at age 7 years. Developmental Neuropsychology, 33, 205–228. 18473197 10.1080/87565640801982312 PMC2729141

[bibr13-2041669520910167] Camos V. Lagner P. Barrouillet P. (2009). Two maintenance mechanisms of verbal information in working memory. Journal of Memory and Language, 61, 457–469.

[bibr14-2041669520910167] Carlson R. A. Sullivan M. A. Schneider W. (1989). Practice and working memory effects in building procedural skill. Journal of Experimental Psychology, 15, 517–526.

[bibr15-2041669520910167] Case R. Kurland D. M. Goldberg J. (1982). Operational efficiency and the growth of short-term memory span. Journal of Experimental Psychology, 33, 386–404.

[bibr16-2041669520910167] Cassimatis N. L. Bello P. Langley P. (2010). Ability, breadth, and parsimony in computational models of higher-order cognition. Cognitive Science, 32, 1304–1322. 10.1080/03640210802455175 21585455

[bibr17-2041669520910167] Chi C. F. Dewi R. S. (2014). Matching performance of vehicle icons in graphical and textual formats. Applied Ergonomics, 45, 904–916. 24315463 10.1016/j.apergo.2013.11.009

[bibr18-2041669520910167] Clark S. E. (1992). Word frequency effects in associative and item recognition. Memory & Cognition, 20, 231–243. 1508049 10.3758/bf03199660

[bibr20-2041669520910167] Cowan N. Ricker T. J. Clark K. M. Hinrichs G. A. Glass B. A. (2015). Knowledge cannot explain the developmental growth of working memory capacity. Developmental Science, 18, 132–145. 24942111 10.1111/desc.12197 PMC4270959

[bibr21-2041669520910167] Cowan N. Rouder J. N. Blume C. L. Saults J. S. (2012). Models of verbal working memory capacity: What does it take to make them work? Psychological Review, 119, 480–499. 22486726 10.1037/a0027791 PMC3618891

[bibr22-2041669520910167] Cox G. E. Hemmer P. Aue W. R. Criss A. H. (2018). Information and processes underlying semantic and episodic memory across tasks, items, and individuals. Journal of Experimental Psychology: General, 147, 545–590. 29698028 10.1037/xge0000407

[bibr23-2041669520910167] Duncan J. Humphreys G. W. (1989). Visual search and stimulus similarity. Psychological Review, 96, 433–458. 2756067 10.1037/0033-295x.96.3.433

[bibr24-2041669520910167] Epelboim J. Steinman R. M. Kowler E. Edwards M. Pizlo Z. Erkelens C. J. Collewijn H. (1995). The function of visual search and memory in sequential looking tasks. Vision Research, 35, 3401–3422. 8560808 10.1016/0042-6989(95)00080-x

[bibr25-2041669520910167] Fung W. Swanson H. L. (2017). Working memory components that predict word problem solving: Is it merely a function of reading, calculation, and fluid intelligence? Memory & Cognition, 45, 1–20. 28378297 10.3758/s13421-017-0697-0

[bibr26-2041669520910167] Gittins D. (1986). Icon-based human-computer interaction. International Journal of Man-Machine Studies, 24, 519–543.

[bibr27-2041669520910167] Goonetilleke R. S. Shih H. M. On H. K. Fritsch J. (2001). Effects of training and representational characteristics in icon design. International Journal of Human-Computer Studies, 55, 741–760.

[bibr28-2041669520910167] Green, A. J. K. & Barnard, P. J. (1990). Iconic interfacing: the role of icon distinctiveness and fixed or variable screen locations. In: Diaper, D., Gilmore, D., Cockton, G., Shackel, B. (Eds.), *Human Computer Interaction – Interact '90*. Elsevier Science Publishers, Amsterdam, pp. 457–462.

[bibr76-2041669520910167] Halford, G. S., Cowan, N. & Andrews, G. (2007). Separating cognitive capacity from knowledge: A new hypothesis. *Trends in Cognitive Sciences*, *11*, 236–242. 10.1016/j.tics.2007.04.001 PMC2613182 17475538

[bibr29-2041669520910167] Horton, W. K.(1994). The icon book: *Visual symbols for computer systems and documentation*. New York: Wiley.

[bibr30-2041669520910167] Huang K. C. (2008). Effects of computer icons and figure/background area ratios and color combinations on visual search performance on an LCD monitor. Displays, 29, 237–242.

[bibr31-2041669520910167] Huang S.-C. Bias R. G. Schnyer D. (2015). How are icons processed by the brain? Neuroimaging measures of four types of visual stimuli used in information systems. Journal of the Association for Information Science & Technology, 66, 702–720.

[bibr32-2041669520910167] Hurlstone M. J. Hitch G. J. Baddeley A. D. (2014). Memory for serial order across domains: An overview of the literature and directions for future research. Psychological Bulletin, 140, 339–373. 24079725 10.1037/a0034221

[bibr33-2041669520910167] Isherwood S. J. Mcdougall S. J. P. Curry M. B. (2007). Icon identification in context: The changing role of icon characteristics with user experience. Human Factors, 49, 465–476. 17552310 10.1518/001872007X200102

[bibr34-2041669520910167] Jaeger T. F. (2008). Categorical data analysis: Away from ANOVAs (transformation or not) and towards logit mixed models. Journal of Memory and Language, 59, 434–446. 19884961 10.1016/j.jml.2007.11.007 PMC2613284

[bibr35-2041669520910167] Lépine R. Barrouillet P. Camos V. (2005). What makes working memory spans so predictive of high-level cognition? Psychonomic Bulletin & Review, 12, 165–170. 15945209 10.3758/bf03196363

[bibr36-2041669520910167] Lewandowsky S. Farrell S. (2008). Short-term memory: New data and a model. Psychology of Learning & Motivation, 49, 1–48.

[bibr37-2041669520910167] Li R. Chen Y. V. Sha C. Lu Z. (2017). Effects of interface layout on the usability of in-vehicle information systems and driving safety. Displays, 49, 123–132.

[bibr38-2041669520910167] Logie R. H. (2011). The functional organization and capacity limits of working memory. Current Directions in Psychological Science, 20, 240–245.

[bibr39-2041669520910167] Luck S. J. Vogel E. K. (2013). Visual working memory capacity: From psychophysics and neurobiology to individual differences. Trends in Cognitive Sciences, 17, 391–400. 23850263 10.1016/j.tics.2013.06.006 PMC3729738

[bibr40-2041669520910167] Ma X. Matta N. Cahier J. Qin C. Cheng Y. (2015). From action icon to knowledge icon: Objective-oriented icon taxonomy in computer science. Displays, 39, 68–79.

[bibr41-2041669520910167] Ma W. J. Husain M. Bays P. M. (2014). Changing concepts of working memory. Nature Neuroscience, 17, 347–356. 24569831 10.1038/nn.3655 PMC4159388

[bibr42-2041669520910167] MacLeod C. M. Kampe K. E. (1996). Word frequency effects on recall, recognition, and word fragment completion tests. Journal of Experimental Psychology: Learning, Memory, and Cognition, 22, 132–142. 10.1037//0278-7393.22.1.132 8648282

[bibr43-2041669520910167] McDougall S. J. P. Curry M. B. de Bruijin O. D. (1999). Measuring symbol and icon characteristics: Norms for concreteness, complexity, meaningfulness, familiarity, and semantic distance for 239 symbols. Behavior Research Methods Instruments & Computers, 31, 487–519. 10.3758/bf03200730 10502873

[bibr44-2041669520910167] McDougall S. J. P. Curry M. B. de Bruijin O. D. (2001). The effects of visual information on users’ mental models: An evaluation of pathfinder analysis as a measure of icon usability. International Journal of Cognitive Ergonomics, 5, 59–84.

[bibr45-2041669520910167] McDougall S. J. P. de Bruijin O. Curry M. B. (2000). Exploring the effects of icon characteristics on user performance: The role of icon concreteness, complexity, and distinctiveness. Journal of Experimental Psychology Applied, 6, 291–306. 11218339 10.1037//1076-898x.6.4.291

[bibr46-2041669520910167] McDougall S. J. P. Reppa I. Kulik J. Taylor A. (2016). What makes icons appealing? The role of processing fluency in predicting icon appeal in different task contexts. Applied Ergonomics, 55, 156–172. 26995046 10.1016/j.apergo.2016.02.006

[bibr47-2041669520910167] McDougall S. J. P. Tyrer V. Folkard S. (2006). Searching for signs, symbols, and icons: Effects of time of day, visual complexity, and grouping. Journal of Experimental Psychology 12, 118–128. 16802893 10.1037/1076-898X.12.2.118

[bibr48-2041669520910167] Muter P. Mayson C. (1986). The role of graphics in item selection from menus. Behaviour & Information Technology, 5, 89–95.

[bibr49-2041669520910167] Nelson A. B. Shiffrin R. M. (2013). The co-evolution of knowledge and event memory. Psychological Review, 120, 356–394. 23458086 10.1037/a0032020

[bibr50-2041669520910167] Oberauer K. Farrell S. Jarrold C. Lewandowsky S. (2016). What limits working memory capacity? Psychological Bulletin, 142, 758–799. 26950009 10.1037/bul0000046

[bibr51-2041669520910167] Oberauer K. Kliegl R. (2006). A formal model of capacity limits in working memory. Journal of Memory & Language, 55, 601–626.

[bibr77-2041669520910167] Oberauer, K. (2009). Design for a working memory. Psychology of Learning and Motivation: *Advances in Research and Theory*, *51*, 45–100.

[bibr52-2041669520910167] Oberauer K. Lewandowsky S. Farrell S. Jarrold C. Greaves M. (2012). Modeling working memory: An interference model of complex span. Psychonomic Bulletin & Review, 19, 779–819. 22715024 10.3758/s13423-012-0272-4

[bibr53-2041669520910167] Quinlan T. P. (2003). Visual feature integration theory: Past, present, and future. Psychological Bulletin, 129, 643–673. 12956538 10.1037/0033-2909.129.5.643

[bibr54-2041669520910167] Rogers Y. (1989). Icon design for the user interface. International Reviews of Ergonomics, 2, 129–155.

[bibr55-2041669520910167] Salman Y. B. Cheng H. I. Patterson P. E. (2012). Icon and user interface design for emergency medical information systems: A case study. International Journal of Medical Informatics, 81, 29–35. 21920810 10.1016/j.ijmedinf.2011.08.005

[bibr56-2041669520910167] Salthouse T. A. (1992). Why do adult age differences increase with task complexity? Developmental Psychology, 28, 905–918.

[bibr57-2041669520910167] Schweickert R. Boruff B. (1986). Short-term memory capacity: Magic number or magic spell? Journal of Experimental Psychology Learning Memory & Cognition, 12, 419–425. 10.1037//0278-7393.12.3.419 2942626

[bibr58-2041669520910167] Shen Z. Xue C. Wang H. (2018). Effects of users’ familiarity with the objects depicted in icons on the cognitive performance of icon identification. i-Perception, *9*(3), 1–17. DOI: 10.1177/2041669518780807. 10.1177/2041669518780807 PMC6024531 29977490

[bibr59-2041669520910167] Siedenburg K. McAdams S. (2017). The role of long-term familiarity and attentional maintenance in short-term memory for timbre. Memory, 25, 550–564. 27314886 10.1080/09658211.2016.1197945

[bibr60-2041669520910167] Silvennoinen J. M. Kujala T. Jokinen J. (2017). Semantic distance as a critical factor in icon design for in-car infotainment systems. Applied Ergonomics, 65, 369–381. 28802458 10.1016/j.apergo.2017.07.014

[bibr61-2041669520910167] SriLakshmi P. Das N. L. Manikumar C. (2017). Low-cost wireless instrumentation for monitoring humidity, wind speed, and direction. Instrumentation Science & Technology, 45, 479–485.

[bibr62-2041669520910167] Stammers R. Hoffman J. (1991). Transfer between icon sets and ratings of icon concreteness and appropriateness. Human Factors & Ergonomics Society Annual Meeting Proceedings, 35, 354–358.

[bibr63-2041669520910167] Stevens C. J. Brennan D. Petocz A. Howell C. (2009). Designing informative warning signals: Effects of indicator type, modality, and task demand on recognition speed and accuracy. Advances in Cognitive Psychology, 5, 84–90. 20523852 10.2478/v10053-008-0064-6 PMC2865004

[bibr64-2041669520910167] Stotts D. B. (1998). The usefulness of icons on the computer interface: Effect of graphical abstraction and functional representation on experienced and novice users. Proceedings of the Human Factors & Ergonomics Society Annual Meeting, 42, 453–457.

[bibr65-2041669520910167] Swanson H. L. Jerman O. Zheng X. (2004). Growth in working memory and mathematical problem solving in children at risk and not at risk for serious math difficulties. Journal of Educational Psychology, 100, 343–379.

[bibr66-2041669520910167] Treisman A. (2006). How the deployment of attention determines what we see. Visual Cognition, 14, 411–443. 17387378 10.1080/13506280500195250 PMC1832144

[bibr67-2041669520910167] Treisman A. Gelade G. (1980). A feature-integration theory of attention. Cognitive Psychology, 12, 97–136. 7351125 10.1016/0010-0285(80)90005-5

[bibr68-2041669520910167] Võ M. L.-H. Wolfe J. M. (2013). The interplay of episodic and semantic memory in guiding repeated search in scenes. Cognition, 126, 198–212. 23177141 10.1016/j.cognition.2012.09.017 PMC3928147

[bibr69-2041669520910167] Wolfe J. M. (2007). *Guided search 4.0: Current progress with a model of visual search*. In W. D. Gray (Ed.), Series on cognitive models and architectures. Integrated models of cognitive systems (pp. 99–119). Oxford University Press.

[bibr70-2041669520910167] Wolfe J. M. Cave K. R. Franzel S. L. (1989). Guided search: An alternative to the feature integration model for visual search. Journal of Experimental Psychology: Human Perception and Performance, 15, 419–433. 2527952 10.1037//0096-1523.15.3.419

[bibr71-2041669520910167] Wolfe J. M. Horowitz T. S. (2004). What attributes guide the deployment of visual attention and how do they do it? Nature Reviews Neuroscience, 5, 495–501. 15152199 10.1038/nrn1411

[bibr72-2041669520910167] Wolfe J. M. Võ M. L.-H. Evans K. K. Greene M. R. (2011). Visual search in scenes involves selective and non-selective pathways. Trends in Cognitive Sciences, 15, 77–84. 21227734 10.1016/j.tics.2010.12.001 PMC3035167

[bibr73-2041669520910167] Xu J. Yue S. (2014). Mimicking visual searching with integrated top down cues and low-level features. Neurocomputing, 133, 1–17.

[bibr74-2041669520910167] Ziefle M. Schröder S. (2006). Icon design on small screens: Effects of miniaturization on speed and accuracy in visual search. Human Factors & Ergonomics Society Annual Meeting Proceedings, 50, 656–660.

