# CORRIGENDUM to “Icon Familiarity Affects the Performance of Complex Cognitive Tasks”

**DOI:** 10.1177/2041669520933027

**Published:** 2020-05-28

Shen, Z., Zhang, L., Xiao, X., Li, R., & Liang, R. (2020). Icon familiarity affects the performance of complex cognitive tasks. *i-Perception*, *11*(2), 1–18. https://doi.org/10.1177/2041669520910167

The authors regret that the following admission was erroneously not included in the article. The experimental paradigm used in this study was developed in Professor Lynne Reder’s lab, as previously published in Shen, Popov, Delahay & Reder (2018). The study’s methods and design were based on those ideas. The following reference should have been included for transparency:

Shen, Z., Popov, V., Delahay, A. B., & Reder, L. M. (2018). Item strength affects working memory capacity. *Memory & Cognition*, *46*(2), 204–215. https://doi.org/10.3758/s13421-017-0758-4

The authors apologise for this omission.

